# EWS/FLI Mediates Transcriptional Repression via NKX2.2 during Oncogenic Transformation in Ewing**'**s Sarcoma

**DOI:** 10.1371/journal.pone.0001965

**Published:** 2008-04-16

**Authors:** Leah A. Owen, Ashley A. Kowalewski, Stephen L. Lessnick

**Affiliations:** 1 Department of Oncological Sciences, University of Utah, Salt Lake City, Utah, United States of America; 2 School of Medicine, University of Utah, Salt Lake City, Utah, United States of America; 3 Center for Children, Huntsman Cancer Institute, University of Utah, Salt Lake City, Utah, United States of America; 4 Division of Pediatric Hematology/Oncology, University of Utah, Salt Lake City, Utah, United States of America; National Cancer Institute at Frederick, United States of America

## Abstract

EWS/FLI is a master regulator of Ewing's sarcoma formation. Gene expression studies in A673 Ewing's sarcoma cells have demonstrated that EWS/FLI downregulates more genes than it upregulates, suggesting that EWS/FLI, and/or its targets, function as transcriptional repressors. One critical EWS/FLI target, NKX2.2, is a transcription factor that contains both transcriptional activation and transcriptional repression domains, raising the possibility that it mediates portions of the EWS/FLI transcriptional signature. We now report that microarray analysis demonstrated that the transcriptional profile of NKX2.2 consists solely of downregulated genes, and overlaps with the EWS/FLI downregulated signature, suggesting that NKX2.2 mediates oncogenic transformation via transcriptional repression. Structure-function analysis revealed that the DNA binding and repressor domains in NKX2.2 are required for oncogenesis in Ewing's sarcoma cells, while the transcriptional activation domain is completely dispensable. Furthermore, blockade of TLE or HDAC function, two protein families thought to mediate the repressive function of NKX2.2, inhibited the transformed phenotype and reversed the NKX2.2 transcriptional profile in Ewing's sarcoma cells. Whole genome localization studies (ChIP-chip) revealed that a significant portion of the NKX2.2-repressed gene expression signature was directly mediated by NKX2.2 binding. These data demonstrate that the transcriptional repressive function of NKX2.2 is necessary, and sufficient, for the oncogenic phenotype of Ewing's sarcoma, and suggest a therapeutic approach to this disease.

## Introduction

Ewing's sarcoma is an aggressive bone and soft tissue tumor of adolescents and young adults [1]. The treatment of this disease involves multimodal therapy and is associated with significant morbidity and mortality. Even with intensive therapies, overall cure rates are approximately 50% at 5 years [2]. More effective, and less toxic, therapies are needed, and are likely to be identified through an improved understanding of the biology of the disease [3].

A recurrent somatic chromosomal translocation, t(11;22)(q24;q12), is present in approximately 85% of Ewing's sarcoma cases, and encodes the EWS/FLI fusion protein [4], [5]. EWS/FLI expression is necessary for the oncogenic phenotype of Ewing's sarcoma cells, and is sufficient to mediate oncogenic transformation of heterologous NIH3T3 cells [6], [7], [8]. Approaches targeting EWS/FLI have been shown to be effective against Ewing's sarcoma in preclinical models [6], [8], [9], [10], [11]. However, only one of these approaches is currently in clinical trials in humans with the disease [11].

EWS/FLI contains a carboxy-terminal ETS-family DNA binding domain contributed by the FLI portion, and an amino-terminal domain contributed by EWS [4]. The EWS portion functions as a strong transcriptional activation domain, and is required for transformation in heterologous NIH3T3 immortalized mouse embryo fibroblasts [7], [12]. Indeed, in this heterologous system, engineered proteins in which the EWS domain in EWS/FLI is replaced with other strong transcriptional activation domains are also oncogenic [13]. These data suggest that EWS/FLI functions as a transcriptional activator to mediate oncogenesis in Ewing's sarcoma. In contrast to the heterologous cell data, however, analysis of the EWS/FLI transcriptional profile in A673 Ewing's sarcoma cells revealed that the fusion protein downregulated more genes than it upregulated [8], [14], [15].

**Figure 1:**
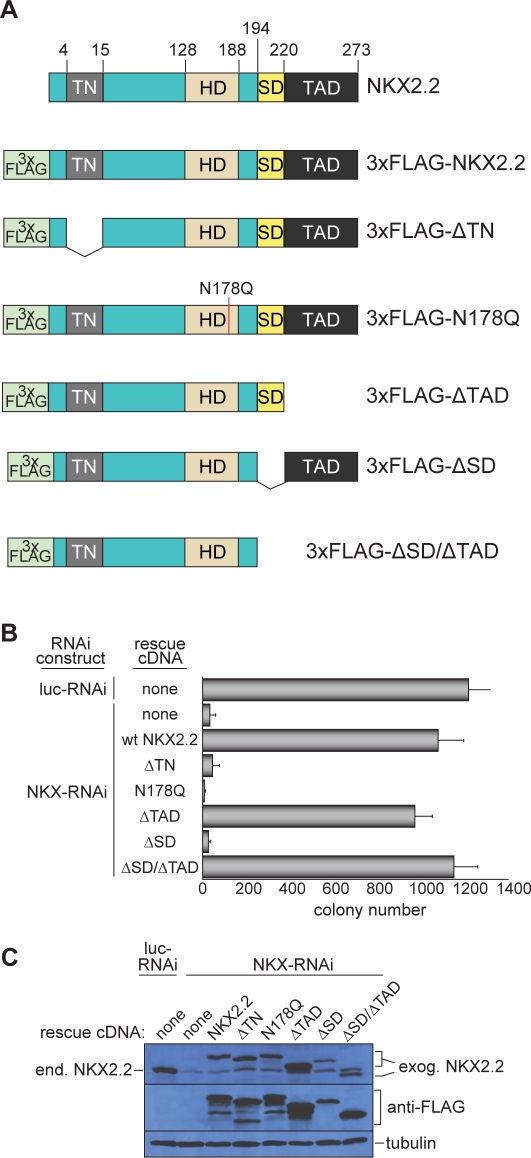
Transcriptional repression and DNA binding domains are required for NKX2.2-mediated Ewing's sarcoma cell oncogenic transformation. (A) Schematic of wild type and 3x-FLAG tagged NKX2.2 constructs. The positions of the transcriptional repressor domain (TN), the homeodomain (HD), the NK2-specific domain (SD), and the transcriptional activation domain (TAD) are shown. (B) Soft agar colony formation of A673 cells infected with the indicated RNAi and cDNA constructs. Error bars indicate standard deviations of duplicate assays. (C) Western blot analysis of A673 cells infected with the indicated RNAi and cDNA constructs, using anti-NKX2.2 antibody (to determine the expression of endogenous NKX2.2 following knockdown using the NKX-RNAi construct), anti-FLAG (to assess the expression of cDNA constructs), or anti-tubulin (as a loading control). The positions of endogenous NKX2.2 (end. NKX2.2) and of the exogenous NKX2.2 constructs (exog. NKX2.2) are indicated.

We recently demonstrated that expression of the transcription factor NKX2.2 is upregulated by EWS/FLI in Ewing's sarcoma and is required for the oncogenic phenotype of the disease [8], [15], [16], [17]. In addition to its DNA binding homeodomain (HD), NKX2.2 harbors both transcriptional activation and repression domains, the presence of which suggests that NKX2.2 acts as a transcriptional activator in some contexts, and as a transcriptional repressor in others (Figure 1A; refs. 18,19). Because a role for NKX2.2 in oncogenesis has only recently been reported, we now report on its molecular mechanism in Ewing's sarcoma development.

## Results and Discussion

To determine the mechanism by which NKX2.2 contributes to oncogenic transformation in Ewing's sarcoma, we analyzed a series of NKX2.2 mutants (Figure 1A) using a “knockdown/rescue” approach. Endogenous NKX2.2 was knocked down in either A673 or SK-N-MC patient-derived Ewing's sarcoma cells using a retrovirally-encoded short hairpin RNA (shRNA) directed against the 3′ UTR of the transcript (NKX-RNAi; ref. 8). Wild type or mutant NKX2.2 cDNAs containing 3xFLAG epitope tags were introduced using retroviral vectors. These cDNAs did not contain the endogenous 3′ UTR, and thus were unaffected by the shRNA. Knockdown of endogenous NKX2.2 results in a severe diminution of oncogenic transformation (Figure 1B; ref. 8). Expression of 3xFLAG wild-type NKX2.2 rescued the loss of transformation (Figure 1B) as efficiently as non-tagged wild-type protein (data not shown).

We found that introduction of a point mutation (N178Q) into the HD DNA binding domain, deletion of the transcriptional repression domain (ΔTN), or deletion of the specific domain (ΔSD), each resulted in a loss of oncogenic rescue activity following knockdown of endogenous NKX2.2. However, a mutant lacking the transcriptional activation domain (ΔTAD) completely rescued transformation. All mutants were expressed at levels equal to the wild-type protein (Figure 1C), and were appropriately localized to the nucleus (Figure S1A). In addition, we found that all mutant constructs maintained their ability to bind DNA, except the N178Q DNA binding domain mutant which showed significantly reduced DNA binding as demonstrated by electrophoretic mobility shift assays (EMSA; Figure S1B; ref. 18). Importantly, we observed no significant differences in growth characteristics of cells expressing these NKX2.2 mutants in comparison to wild-type controls (Figure S1C). Based on these data, the TN, HD and SD domains of NKX2.2 are required for oncogenic transformation in Ewing's sarcoma, while the TAD domain is dispensable.

The importance of the transcriptional repression and DNA binding domains suggests a critical role for NKX2.2-mediated transcriptional repression in the pathogenesis of Ewing's sarcoma. However, the additional importance of the specific domain (SD) for transformation was more difficult to reconcile with this model of NKX2.2 function. The SD lies adjacent to the TAD and blocks its function (Figure 1A; ref. 18). Removal of the SD then allows for expression of the transcriptional activation function of TAD. Therefore, we hypothesized that NKX2.2-mediated transcriptional activation is deleterious to oncogenic transformation. In support of this hypothesis, additional deletion of TAD when the SD is absent results in a mutant (ΔSD/ΔTAD; Figure 1A) that has full oncogenic function (Figure 1B).

**Figure 2:**
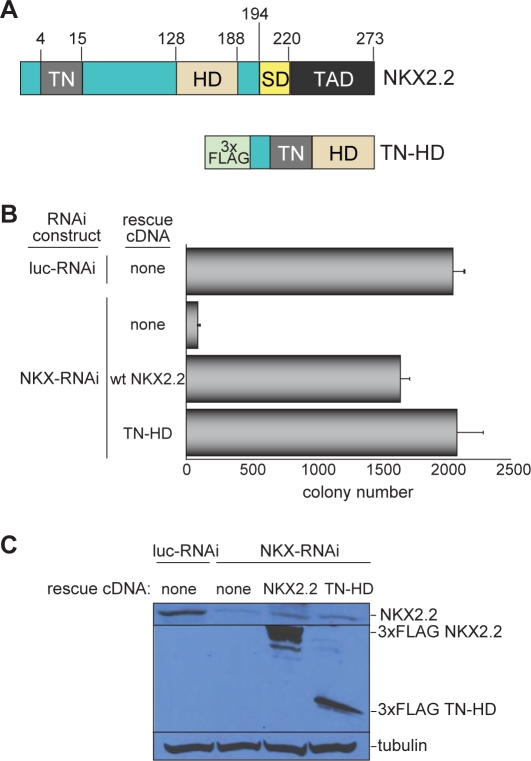
The TN and HD domains are sufficient to rescue NKX2.2-mediated Ewing's sarcoma oncogenic transformation. (A) Schematic diagram of the 3xFLAG-tagged TN-HD fusion protein. (B) Soft agar colony formation of A673 cells infected with the indicated RNAi and cDNA constructs demonstrate that the TN-HD fusion rescues oncogenic transformation as efficiently as wild-type (wt) NKX2.2. Error bars indicate standard deviations of duplicate assays. (C) Western blot analysis of A673 cells infected with either control luc-RNAi, or NKX-RNAi, constructs, and the TN-HD fusion, or wild-type NKX2.2, using anti-NKX2.2 antibody (to determine the expression of endogenous NKX2.2 following knockdown using the NKX-RNAi construct), anti-FLAG (to assess the expression of cDNA constructs), or anti-tubulin (as a loading control).

We next asked if the TN transcriptional repression domain was sufficient for NKX2.2-mediated transformation. An NKX2.2 mutant construct was developed containing only the TN and HD domains (TN-HD fusion; Figure 2A). This mutant was fully functional in the oncogenic transformation rescue assay (Figure 2B). As with the other NKX2.2 mutant constructs, TN-HD was expressed (Figure 2C), localized to the nucleus, bound DNA appropriately, and displayed normal growth in tissue culture (Figure S2A-S2C). Thus, the DNA binding and transcriptional repression domains are necessary and sufficient for the oncogenic function of NKX2.2.

EWS/FLI downregulates a greater number of genes than it upregulates [8], [14]. The structure-function data presented here raises the possibility that at least a portion of the EWS/FLI downregulated signature is indirectly mediated by NKX2.2. To test this hypothesis explicitly, we determined the transcriptional profile of NKX2.2. Triplicate polyclonal populations of A673 Ewing's sarcoma cells were prepared harboring the NKX-RNAi retroviral construct, or a control luc-RNAi construct. An additional set of cells was prepared which contained the NKX-RNAi knockdown construct with either the wild-type 3xFLAG-tagged NKX2.2 cDNA, or an empty vector control. Thus, two classes of samples were defined: (1) “NKX2.2-expressed” (luc-RNAi and NKX-RNAi/NKX2.2 cDNA rescue cells) and (2) “NKX2.2-knockdown” (NKX-RNAi and NKX-RNAi/“empty-vector” rescue). The transcriptional profile was determined using Affymetrix U133plus2 microarrays. The signal-to-noise metric and permutation testing at the 99% confidence level were used to identify genes whose expression depended on NKX2.2. Consistent with our hypothesis that it functions as a transcriptional repressor, we found that NKX2.2 downregulated 159 probesets (i.e., genes), but none were upregulated by the protein.

To determine whether the NKX2.2 signature contributes to the EWS/FLI downregulated transcriptional profile, we compared these data. To facilitate a direct comparison between these profiles using the same microarray platform, we prepared A673 Ewing's sarcoma cells consisting of “EWS/FLI-expressed” (luc-RNAi and EF-2-RNAi/EWS/FLI cDNA rescue cells) or “EWS/FLI-knockdown” (EF-2-RNAi and EF-2-RNAi/“empty-vector” rescue) classes, and analyzed them on Affymetrix U133plus2 microarrays. As with the NKX2.2 dataset, we used the signal-to-noise metric and permutation testing at the 99% confidence level. We found that only 469 genes were upregulated by EWS/FLI, while 3075 genes were downregulated. This ratio of up- to downregulated genes was similar to what has been previously published, and therefore provides an independent validation of the data we, and others, have reported [8], [14].

**Figure 3:**
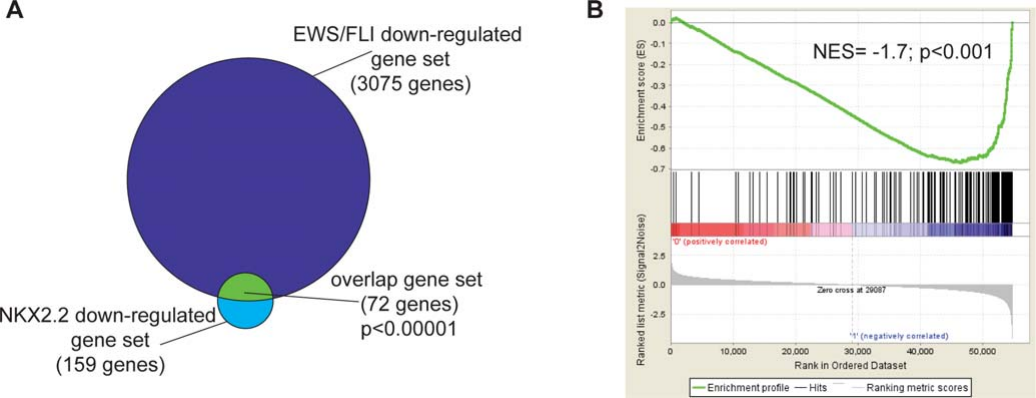
NKX2.2 downregulated target genes are enriched in the EWS/FLI downregulated dataset. (A) Venn diagram representation of the overlap between EWS/FLI downregulated genes and NKX2.2 downregulated genes. The Chi square-determined p-value is indicated. (B) Gene set enrichment analysis (GSEA) using EWS/FLI-regulated genes as the rank-ordered dataset and NKX2.2 downregulated genes as the geneset. EWS/FLI-regulated genes are shown on the x-axis, with upregulated genes toward the left, and downregulated genes toward the right. The positions of genes that were downregulated by NKX2.2 are indicated by the black vertical lines in the center portion of the panel. Ranking metric scores are shown in the bottom portion of the panel. The normalized enrichment score (NES) and p-value are shown.

Chi square analysis was used to compare the NKX2.2 and EWS/FLI datasets. We found that 72 of the 159 NKX2.2 downregulated genes were also downregulated by EWS/FLI (p<0.00001; Figure 3A, Table S1). To evaluate these data using a different statistical approach, gene set enrichment analysis (GSEA; ref. 20) was used to compare these datasets. GSEA measures the enrichment of one gene list against a second rank-ordered dataset using a running sum statistic called the normalized enrichment score. This analysis demonstrated that EWS/FLI downregulated genes were highly enriched for the NKX2.2 downregulated geneset (NES = −1.7; p<0.001; Figure 3B). Thus, both approaches demonstrate that NKX2.2 mediates a statistically significant portion of the EWS/FLI downregulated transcriptional signature. These data suggest a model whereby EWS/FLI upregulates NKX2.2, which then mediates a subset of the EWS/FLI downregulated transcriptional signature.

**Figure 4:**
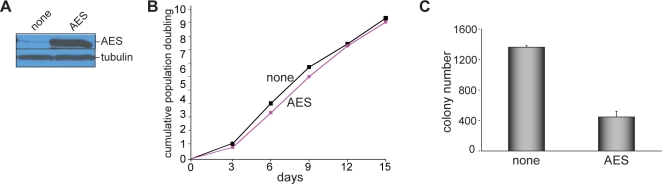
Role of TLE corepressors in NKX2.2-mediated gene repression. (A) Western blot showing retroviral-mediated expression of dominant-negative TLE (AES) in A673 Ewing's sarcoma cells as compared to an empty vector control (“none”). Tubulin is shown as a loading control. (B) Tissue culture growth of A673 Ewing's sarcoma cells expressing AES was unchanged as compared to empty vector control bearing cells (“none”). These data are a representative example from multiple experimental replicates. (C) Colony formation of A673 Ewing's sarcoma cells in soft agar was inhibited by AES expression, as compared to empty vector control infected cells (“none”). Error bars indicate standard deviations of duplicate assays.

We next sought to determine the mechanistic basis for NKX2.2-mediated transcriptional repression in Ewing's sarcoma oncogenesis. During neural tube and pancreatic β-cell development, NKX2.2 functions primarily as a transcriptional repressor through recruitment of TLE corepressors [19], [21]. The transcripts for TLEs 1, 2, 3 and 4 are all expressed in Ewing's sarcoma cell lines (data not shown). To determine if TLE family members participate in Ewing's sarcoma oncogenic transformation, we used a naturally occurring TLE dominant-negative protein, amino-terminal enhancer of split (AES; ref. 22). AES was expressed in SK-N-MC Ewing's sarcoma cells using a retroviral construct (Figure 4A). While AES expression did not affect the tissue culture growth characteristics of the cells, oncogenic transformation was diminished as compared with controls (Figure 4B–4C). These data suggest that TLE activity is required for Ewing's sarcoma oncogenic transformation, supporting its role as a corepressor for NKX2.2 in this disease.

**Figure 5:**
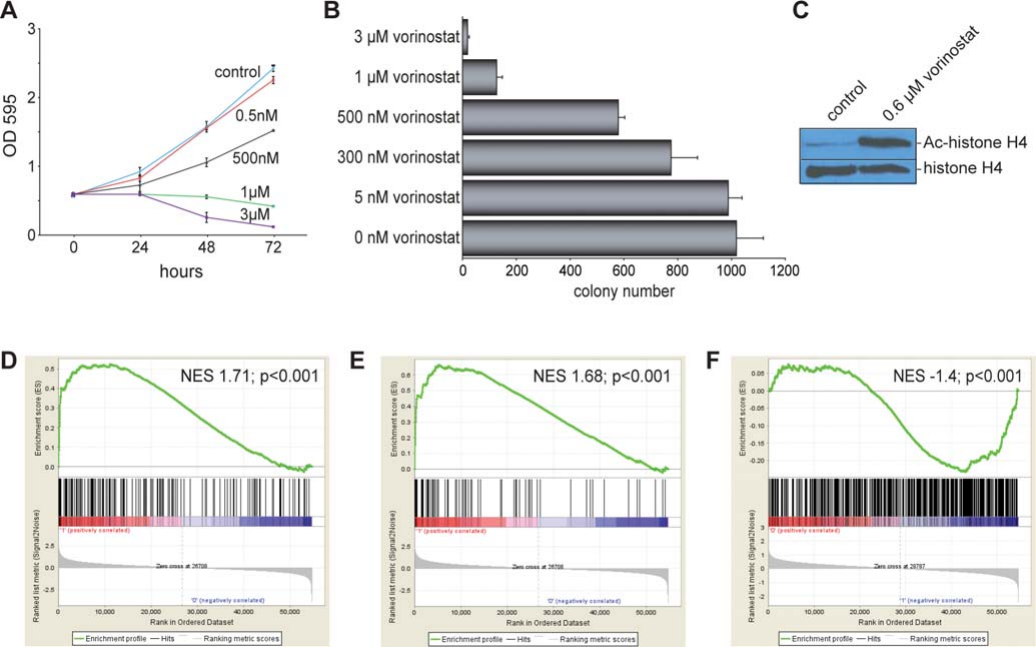
HDACs mediate repression of NKX2.2 targets. (A) Inclusion of the indicated concentrations of vorinostat inhibited the short-term tissue culture growth of A673 Ewing's sarcoma cells in a dose-dependent manner. The OD595 of crystal violet staining is shown on the y-axis, and is proportional to cell number. (B) Addition of the indicated concentrations of the HDAC inhibitor vorinostat caused a dose-dependent decrease in soft agar colony formation of A673 Ewing's sarcoma cells. Error bars indicate standard deviations of duplicate assays. (C) 0.6 µM of vorinostat is sufficient to cause an increase of acetylated histone H4 in A673 Ewing's sarcoma cells, as observed by Western blot, as compared to vehicle control. Total histone H4 levels are shown as a loading control. (D) Gene set enrichment analysis (GSEA) using vorinostat-regulated genes in A673 Ewing's sarcoma cells as the rank-ordered dataset and NKX2.2 downregulated genes as the geneset. Vorinostat-regulated genes are shown on the x-axis, with upregulated genes toward the left, and downregulated genes toward the right. The positions of genes that were downregulated by NKX2.2 are indicated by the black vertical lines in the center portion of the panel. Ranking metric scores are shown in the bottom portion of the panel. The normalized enrichment score (NES) and p-value are shown. (E) Gene set enrichment analysis (GSEA) using vorinostat-regulated genes in A673 Ewing's sarcoma cells as the rank-ordered dataset and the 72 genes downregulated by both EWS/FLI and NKX2.2 as the geneset. The normalized enrichment score (NES) and p-value are shown. (F) Gene set enrichment analysis (GSEA) using NKX2.2-regulated genes as the rank-ordered dataset and NKX2.2 ChIP-chip targets as the geneset. The normalized enrichment score (NES) and p-value are shown.

TLE family members mediate transcriptional repression, in part, through recruitment of histone deacetylases (HDACs; ref. 23). Indeed, HDACs are recruited to NKX2.2-dependent promoters involved in oligodendrocyte development [24]. To determine if NKX2.2-mediated oncogenic transformation requires HDAC function, we used a clinically-approved HDAC inhibitor, vorinostat (Zolinza™, formerly suberoylanilide hydroxamic acid, SAHA), in transformation assays. Inclusion of vorinostat effectively prevented Ewing's sarcoma cell growth in tissue culture, and colony formation in soft-agar, in a dose-dependent manner (Figure 5A–5B). These data complement recently published work demonstrating that multiple HDAC inhibitors are effective against Ewing's sarcoma in preclinical studies including murine xenografts [25], [26], [27].

HDAC inhibitors are likely to block oncogenic transformation through multiple transcriptional repressors, and evidence of their efficacy is not sufficient to suggest that they mediate their activities by blocking NKX2.2 function. To address this question directly, we analyzed the transcriptional profile of A673 Ewing's sarcoma cells treated with a concentration of vorinostat sufficient to induce histone acetylation, yet minimally affect growth in tissue culture (Figure 5C and data not shown).

We used GSEA to compare the transcriptional profiles of A673 Ewing's sarcoma cells treated with vorinostat to the same cells in which NKX2.2 was knocked down. The gene expression data from vorinostat-treated cells were rank ordered using the signal-to-noise statistic. The 159 genes downregulated by NKX2.2 were found to be enriched among the genes that were upregulated by vorinostat (NES = 1.71; p<0.001; Figure 5D). Thus, vorinostat reverses the NKX2.2-mediated transcriptional signature. Similarly, when this analysis was performed using the 72 genes found to be downregulated by both EWS/FLI and NKX2.2, we found these genes to cluster equally strongly with genes that were upregulated by vorinostat (NES = 1.68; p<0.001; Figure 5E). This strongly suggests that HDAC-mediated gene repression is involved in the transcriptional function of NKX2.2 in Ewing's sarcoma.

Taken together, these data suggest that NKX2.2 mediates repression of its targets by directly binding to their promoters. To test this hypothesis, we performed whole genome localization studies (ChIP-chip). Antibodies against endogenous NKX2.2 did not function well in our initial experiments. Therefore, we used A673 Ewing's cells in which endogenous NKX2.2 was knocked down using the NKX-RNAi construct, and a 3xFLAG-tagged NKX2.2 was subsequently expressed. These cells represent our NKX2.2 “knockdown/rescue” condition, which was fully functional with respect to oncogenic transformation in Ewing's sarcoma (Figure 1B). Cross-linked DNA/protein complexes were immunoprecipitated using an anti-FLAG antibody and recovered DNA was hybridized to Agilent 244k promoter microarrays. Using this approach, we identified approximately 600 genes that were directly bound by NKX2.2 (data not shown). GSEA comparison of directly bound targets with the NKX2.2 knockdown/rescue microarray dataset revealed a statistically significant enrichment of NKX2.2 bound targets with NKX2.2 repressed genes (NES = −1.4; p<0.001; Figure 5F). These data suggest that NKX2.2 represses gene expression through direct binding to promoters and recruitment of TLE and HDAC family members in the majority of its targets. It should be noted that in some cases NKX2.2 could function by competing with the recruitment of positively-regulating transcription factors. Additional work at specific promoters will be required to evaluate this possibility.

Transcriptional repression is central to the pathogenesis of several types of cancers, including both acute myelogenous leukemias (AML) and acute lymphoblastic leukemias (ALL). For example, acute promyelocytic leukemia (APML), is associated with the t(15;17)(q24;q21) encoding the PML-RARα fusion product. PML-RARα acts as a transcriptional repressor to block retinoic acid responsive genes in the absence of ligand due to its interaction with HDACs [28]. All-trans retinoic acid (ATRA) reverses the repressive activity of PML-RARα, and thereby alters its oncogenic function, making it an important therapeutic agent for patients with APML. The addition of HDAC inhibitors in combination with ATRA treatment has shown increased efficacy in preclinical models of this disease [29], [30]. Therefore, transcriptional repressors are an important therapeutic target in hematologic malignancies.

In contrast, evidence from solid tumor development has largely pointed to an important role for transcriptional activation. For example, the PAX3/FKHR fusion in alveolar rhabdomyosarcoma, the TLS/CHOP fusion in myxoid liposarcoma, and the EWS/ATF1 fusion in clear cell sarcoma have all been shown to function as transcriptional activators [31], [32], [33]. Similarly, evidence has also suggested that EWS/FLI functions as a transcriptional activator, and that this is required for its role in oncogenesis [12], [13]. While EWS/FLI-mediated transcriptional activation may be important, our data suggest that transcriptional repression plays a critical role in the pathogenesis of Ewing's sarcoma. This repression is attributable, in part, to the EWS/FLI upregulated target NKX2.2, and is mediated through HDACs. Reversal of the NKX2.2 signature by vorinostat provides correlative data for a role of the protein in the blockade of oncogenic transformation by the drug. These data complement our mechanistic work and support a role for NKX2.2-mediated transcriptional repression in Ewing's sarcoma oncogenesis.

In summary, the EWS/FLI target protein, NKX2.2, functions as a transcriptional repressor in Ewing's sarcoma. This function is both necessary, and sufficient, for its participation in the oncogenic phenotype of the disease. This is an important first step in understanding the transcriptional hierarchy induced by EWS/FLI. For example, NKX2.2 mediates a highly significant portion of the EWS/FLI downregulated signature. However, the absolute number of genes repressed by NKX2.2 is small, consisting of approximately 2% of the EWS/FLI repressed signature. This suggests that additional EWS/FLI targets, or EWS/FLI itself, contribute to the remaining portion of the signature. In addition, the role of HDACs as effectors of transcriptional repression in Ewing's sarcoma suggests that HDAC inhibitors might be a useful therapeutic option for patients with this devastating cancer of children and young adults.

## Materials and Methods

### Constructs and retroviruses

NKX-RNAi, luc-RNAi, and ERG-RNAi were previously described [8]. The 3xFLAG tag (from p3xFLAG-CMV10; Sigma) was introduced onto the amino-terminus of NKX2.2, and its mutants, and the cDNAs were cloned into the pQCXIN retroviral vector (Clontech) using standard techniques.

### Cell Culture

A673 and SK-N-MC Ewing's sarcoma cell lines were grown as described [8], [34]. Following retroviral infection, polyclonal cell populations were prepared by growth in the appropriate selection media (2 µg/ml for puromycin, 300 µg/ml for G418). Soft agar and 3T5 growth assays were performed as described [8]. Crystal violet analysis of cell growth +/− vorinostat was performed by seeding A673 cells at a concentration of 5×10^6^ cells in 10cm tissue culture plates. 12 hours after plating, vorinostat, or vehicle (methanol), was added. At various time points, cells were fixed with 10% buffered formalin, and cell density was quantified by crystal violet staining as described [34].

### Immunodetection

The following antibodies were used: M2-anti-FLAG (Sigma F-1804), anti-α-tubulin (Calbiochem CP06), anti-NKX2.2 (Santa Cruz sc15015), anti-histone H4 (Upstate 07-108), Anti-acetyl-lysine (Upstate 06-933), and anti-AES1 (Imgenex IMG-5408).

### Electrophoretic mobility shift assays (EMSA)

EMSA buffers and electrophoresis conditions were previously described [35]. Nuclear extracts were prepared from 293EBNA cells transfected with 3xFLAG-NKX2.2 expression plasmids, or empty vector control. Two µg of nuclear extract protein and 5 nM [^32^P]-labeled NKX2.2 binding probes [18] were used in each reaction. One hundred fold excess (500 nM) of specific (or mutant) unlabeled competitors were used in the indicated reactions [18].

### Microarray Analysis

RNA was prepared from independent triplicate samples using RNAeasy (Qiagen) and processed for microarray hybridization as previously described [8]. Microarray analysis was conducted using GenePattern 2.0 (http://www.broad.mit.edu/cancer/software/genepattern/). Microarrays were normalized using the MAS5 algorithm, and expression threshold and ceiling values were applied as described [8]. Expression data was filtered for a 3 fold change across samples, with a minimal “delta” value of 50. Samples were rank-ordered using the signal-to-noise statistic, and significant changes were identified using permutation testing with a p-value of 0.01 [8]. Overlaps between different genesets were analyzed using the VennMaster program (http://www.informatik.uni-ulm.de/ni/mitarbeiter/HKestler/vennm/doc.html). Statistical significance of overlaps was determined using Chi square analysis. Gene set enrichment analysis (GSEA) was performed using GSEA1.0 program (http://www.broad.mit.edu/gsea/; ref. 20).

### Whole genome localization studies (ChIP-chip)

ChIP-chip was performed as described [36], except that A673 Ewing's sarcoma cells and the M2-anti-FLAG antibody (Sigma F-1804) were used. Two independent biologic replicates were hybridized to Agilent 244k promoter microarrays. These microarrays interrogate ∼17k human promoters from −5.5 kb to +2.5 kb relative to the transcriptional start site. Initial analysis of the datasets was performed using the Agilent ChIP Analytics software (version 1.3.1) to average both replicates as previously described [36].

## Supporting Information

Table S1 Genes downregulated by both EWS/FLI and NKX2.2
(0.09 MB DOC)

Figure S1 NKX2.2 mutants are localized to the nucleus, bind DNA appropriately, and have growth characteristics similar to control. (A) Immunofluorescence of A673 cells infected with the indicated cDNA constructs with anti-FLAG antibody demonstrate nuclear localization of each of the NKX2.2 mutants. Nuclei are shown by DAPI staining. (B) Electrophoretic mobility shift assays (EMSA) using nuclear extracts from 293EBNA cells transfected with the indicated constructs demonstrate that all of the NKX2.2 constructs, except for the N178Q homeodomain DNA binding mutant, bind a consensus NKX2.2 DNA duplex. Supershifts were performed using anti-FLAG antibody. Specific complexes were competed using wild-type unlabeled competitor, while non-specific complexes were competed using mutant unlabeled competitor. The position of NKX2.2 bound complexes are indicated in the first lane of each group by a red asterisk. (C) Growth analysis using a 3T5 assay demonstrates that A673 cells harboring the NKX2.2 mutant constructs grow similarly to cells expressing the wild type NKX2.2 or empty vector rescue constructs. These data are a representative example from multiple experimental replicates.
(7.61 MB TIF)

Figure S2 The TN-HD mutant is localized to the nucleus, binds DNA, and has growth characteristics similar to control. (A) Immunofluorescence of A673 cells infected with the indicated cDNA constructs with anti-FLAG antibody demonstrate nuclear localization of the TN-HD NKX2.2 mutants. Nuclei are shown by DAPI staining. (B) Electrophoretic mobility shift assays (EMSA) using nuclear extracts from 293EBNA cells transfected with the TN-HD fusion protein demonstrates that the fusion binds a duplex DNA containing an NKX2.2 consensus binding site. Supershift was performed using anti-FLAG antibody. Specific complexes were competed using wild-type unlabeled competitor, while non-specific complexes were competed using mutant unlabeled competitor. The position of the TN-HD-bound complex is indicated in the first lane by a red asterisk. (C) Growth analysis using a 3T5 assay demonstrates that A673 cells harboring the NKX2.2 mutant TN-HD grows similarly to cells expressing the wild type NKX2.2 or empty vector rescue constructs. These data are a representative example from multiple experimental replicates.
(5.39 MB TIF)

## References

[1] Grier HE (1997). The Ewing family of tumors. Ewing's sarcoma and primitive neuroectodermal tumors.. Pediatr Clin North Am.

[2] Rodriguez-Galindo C, Spunt SL, Pappo AS (2003). Treatment of Ewing sarcoma family of tumors: current status and outlook for the future.. Med Pediatr Oncol.

[3] McAllister NR, Lessnick SL (2005). The potential for molecular therapeutic targets in Ewing's sarcoma.. Curr Treat Options Oncol.

[4] Delattre O, Zucman J, Plougastel B, Desmaze C, Melot T (1992). Gene fusion with an ETS DNA-binding domain caused by chromosome translocation in human tumours.. Nature.

[5] Turc-Carel C, Aurias A, Mugneret F, Lizard S, Sidaner I (1988). Chromosomes in Ewing's sarcoma. I. An evaluation of 85 cases of remarkable consistency of t(11;22)(q24;q12).. Cancer Genet Cytogenet.

[6] Kinsey M, Smith R, Lessnick SL (2006). NR0B1 Is Required for the Oncogenic Phenotype Mediated by EWS/FLI in Ewing's Sarcoma.. Mol Cancer Res.

[7] May WA, Gishizky ML, Lessnick SL, Lunsford LB, Lewis BC (1993). Ewing sarcoma 11;22 translocation produces a chimeric transcription factor that requires the DNA-binding domain encoded by FLI1 for transformation.. Proc Natl Acad Sci U S A.

[8] Smith R, Owen LA, Trem DJ, Wong JS, Whangbo JS (2006). Expression profiling of EWS/FLI identifies NKX2.2 as a critical target gene in Ewing's sarcoma.. Cancer Cell.

[9] Hu-Lieskovan S, Heidel JD, Bartlett DW, Davis ME, Triche TJ (2005). Sequence-specific knockdown of EWS-FLI1 by targeted, nonviral delivery of small interfering RNA inhibits tumor growth in a murine model of metastatic Ewing's sarcoma.. Cancer Res.

[10] Lambert G, Bertrand JR, Fattal E, Subra F, Pinto-Alphandary H (2000). EWS fli-1 antisense nanocapsules inhibits Ewing sarcoma-related tumor in mice.. Biochem Biophys Res Commun.

[11] Stegmaier K, Wong JS, Ross KN, Chow KT, Peck D (2007). Signature-based small molecule screening identifies cytosine arabinoside as an EWS/FLI modulator in Ewing sarcoma.. PLoS Med.

[12] May WA, Lessnick SL, Braun BS, Klemsz M, Lewis BC (1993). The Ewing's sarcoma EWS/FLI-1 fusion gene encodes a more potent transcriptional activator and is a more powerful transforming gene than FLI-1.. Mol Cell Biol.

[13] Lessnick SL, Braun BS, Denny CT, May WA (1995). Multiple domains mediate transformation by the Ewing's sarcoma EWS/FLI- 1 fusion gene.. Oncogene.

[14] Prieur A, Tirode F, Cohen P, Delattre O (2004). EWS/FLI-1 silencing and gene profiling of Ewing cells reveal downstream oncogenic pathways and a crucial role for repression of insulin-like growth factor binding protein 3.. Mol Cell Biol.

[15] Owen LA, Lessnick SL (2006). Identification of Target Genes in Their Native Cellular Context: An Analysis of EWS/FLI in Ewing's Sarcoma.. Cell Cycle.

[16] Sussel L, Kalamaras J, Hartigan-O'Connor DJ, Meneses JJ, Pedersen RA (1998). Mice lacking the homeodomain transcription factor Nkx2.2 have diabetes due to arrested differentiation of pancreatic beta cells.. Development.

[17] Briscoe J, Sussel L, Serup P, Hartigan-O'Connor D, Jessell TM (1999). Homeobox gene Nkx2.2 and specification of neuronal identity by graded Sonic hedgehog signaling.. Nature.

[18] Watada H, Mirmira RG, Kalamaras J, German MS (2000). Intramolecular control of transcriptional activity by the NK2-specific domain in NK-2 homeodomain proteins.. Proc Natl Acad Sci U S A.

[19] Muhr J, Andersson E, Persson M, Jessell TM, Ericson J (2001). Groucho-mediated transcriptional repression establishes progenitor cell pattern and neuronal fate in the ventral neural tube.. Cell.

[20] Subramanian A, Tamayo P, Mootha VK, Mukherjee S, Ebert BL (2005). Gene set enrichment analysis: a knowledge-based approach for interpreting genome-wide expression profiles.. Proc Natl Acad Sci U S A.

[21] Doyle MJ, Loomis ZL, Sussel L (2007). Nkx2.2-repressor activity is sufficient to specify alpha-cells and a small number of beta-cells in the pancreatic islet.. Development.

[22] Tetsuka T, Uranishi H, Imai H, Ono T, Sonta S (2000). Inhibition of nuclear factor-kappaB-mediated transcription by association with the amino-terminal enhancer of split, a Groucho-related protein lacking WD40 repeats.. J Biol Chem.

[23] Chen G, Fernandez J, Mische S, Courey AJ (1999). A functional interaction between the histone deacetylase Rpd3 and the corepressor groucho in Drosophila development.. Genes Dev.

[24] Wei Q, Miskimins WK, Miskimins R (2005). Stage-specific expression of myelin basic protein in oligodendrocytes involves Nkx2.2-mediated repression that is relieved by the Sp1 transcription factor.. J Biol Chem.

[25] Sonnemann J, Dreyer L, Hartwig M, Palani CD, Hong LT (2007). Histone deacetylase inhibitors induce cell death and enhance the apoptosis-inducing activity of TRAIL in Ewing's sarcoma cells.. J Cancer Res Clin Oncol.

[26] Sakimura R, Tanaka K, Nakatani F, Matsunobu T, Li X (2005). Antitumor effects of histone deacetylase inhibitor on Ewing's family tumors.. Int J Cancer.

[27] Jaboin J, Wild J, Hamidi H, Khanna C, Kim CJ (2002). MS-27-275, an inhibitor of histone deacetylase, has marked in vitro and in vivo antitumor activity against pediatric solid tumors.. Cancer Res.

[28] Minucci S, Pelicci PG (1999). Retinoid receptors in health and disease: co-regulators and the chromatin connection.. Semin Cell Dev Biol.

[29] Grignani F, De Matteis S, Nervi C, Tomassoni L, Gelmetti V (1998). Fusion proteins of the retinoic acid receptor-alpha recruit histone deacetylase in promyelocytic leukaemia.. Nature.

[30] Guidez F, Ivins S, Zhu J, Soderstrom M, Waxman S (1998). Reduced retinoic acid-sensitivities of nuclear receptor corepressor binding to PML- and PLZF-RARalpha underlie molecular pathogenesis and treatment of acute promyelocytic leukemia.. Blood.

[31] Prasad DD, Ouchida M, Lee L, Rao VN, Reddy ES (1994). TLS/FUS fusion domain of TLS/FUS-erg chimeric protein resulting from the t(16;21) chromosomal translocation in human myeloid leukemia functions as a transcriptional activation domain.. Oncogene.

[32] Sublett JE, Jeon IS, Shapiro DN (1995). The alveolar rhabdomyosarcoma PAX3/FKHR fusion protein is a transcriptional activator.. Oncogene.

[33] Fujimura Y, Ohno T, Siddique H, Lee L, Rao VN (1996). The EWS-ATF-1 gene involved in malignant melanoma of soft parts with t(12;22) chromosome translocation, encodes a constitutive transcriptional activator.. Oncogene.

[34] Lessnick SL, Dacwag CS, Golub TR (2002). The Ewing's sarcoma oncoprotein EWS/FLI induces a p53-dependent growth arrest in primary human fibroblasts.. Cancer Cell.

[35] Smith SB, Ee HC, Conners JR, German MS (1999). Paired-homeodomain transcription factor PAX4 acts as a transcriptional repressor in early pancreatic development.. Mol Cell Biol.

[36] Hollenhorst PC, Shah AA, Hopkins C, Graves BJ (2007). Genome-wide analyses reveal properties of redundant and specific promoter occupancy within the ETS gene family.. Genes Dev.

