# Supplementary material for: EWS/FLI Mediates Transcriptional Repression via NKX2.2 during Oncogenic Transformation in Ewing's Sarcoma
Source: PLoS One. 2008 Apr 16;3(4):e1965. doi: 10.1371/journal.pone.0001965 (PMC2291578; doi:10.1371/journal.pone.0001965)
Supplement: Table S1 — Genes downregulated by both EWS/FLI and NKX2.2 (0.09 MB DOC) [file pone.0001965.s001.doc]

| **Affymetrix Probe ID** | **Gene Symbol** | **Gene Name** |
| --- | --- | --- |
| 1555724_s_at | TAGLN | transgelin |
| 1558692_at | C1orf85 | chromosome 1 open reading frame 85 |
| 200743_s_at | TPP1 | tripeptidyl peptidase I |
| 201289_at | CYR61 | cysteine-rich, angiogenic inducer, 61 |
| 201427_s_at | SEPP1 | selenoprotein P, plasma, 1 |
| 201506_at | TGFBI | transforming growth factor, beta-induced, 68kDa |
| 201852_x_at | COL3A1 | collagen, type III, alpha 1 (Ehlers-Danlos syndrome type IV, autosomal dominant) |
| 202274_at | ACTG2 | actin, gamma 2, smooth muscle, enteric |
| 202404_s_at | COL1A2 | collagen, type I, alpha 2 |
| 202465_at | PCOLCE | procollagen C-endopeptidase enhancer |
| 202723_s_at | FOXO1 | forkhead box O1 |
| 202764_at | STIM1 | stromal interaction molecule 1 |
| 202769_at | CCNG2 | cyclin G2 |
| 203232_s_at | ATXN1 | ataxin 1 |
| 203308_x_at | HPS1 | Hermansky-Pudlak syndrome 1 |
| 203382_s_at | APOE | apolipoprotein E |
| 203739_at | ZNF217 | zinc finger protein 217 |
| 204298_s_at | LOX | lysyl oxidase |
| 204471_at | GAP43 | growth associated protein 43 |
| 204547_at | RAB40B | RAB40B, member RAS oncogene family |
| 204854_at | LEPREL2 | leprecan-like 2 |
| 205151_s_at | KIAA0644 | KIAA0644 gene product |
| 205182_s_at | ZNF324 | zinc finger protein 324 |
| 205547_s_at | TAGLN | transgelin |
| 205889_s_at | JAKMIP2 | janus kinase and microtubule interacting protein 2 |
| 205924_at | RAB3B | RAB3B, member RAS oncogene family |
| 206157_at | PTX3 | pentraxin-related gene, rapidly induced by IL-1 beta |
| 206243_at | TIMP4 | TIMP metallopeptidase inhibitor 4 |
| 208119_s_at | ZNF93 | zinc finger protein 93 |
| 209515_s_at | RAB27A | RAB27A, member RAS oncogene family |
| 209700_x_at | PDE4DIP | phosphodiesterase 4D interacting protein (myomegalin) |
| 209771_x_at | CD24 | CD24 molecule |
| 209955_s_at | FAP | fibroblast activation protein, alpha |
| 210809_s_at | POSTN | periostin, osteoblast specific factor |
| 210869_s_at | MCAM | melanoma cell adhesion molecule |
| 211161_s_at | COL3A1 | collagen, type III, alpha 1 (Ehlers-Danlos syndrome type IV, autosomal dominant) |
| 212286_at | ANKRD12 | ankyrin repeat domain 12 |
| 212358_at | CLIP3 | CAP-GLY domain containing linker protein 3 |
| 212759_s_at | TCF7L2 | transcription factor 7-like 2 (T-cell specific, HMG-box) |
| 213002_at | MARCKS | myristoylated alanine-rich protein kinase C substrate |
| 213024_at | TMF1 | TATA element modulatory factor 1 |
| 213758_at | COX4I1 | cytochrome c oxidase subunit IV isoform 1 |
| 215076_s_at | COL3A1 | collagen, type III, alpha 1 (Ehlers-Danlos syndrome type IV, autosomal dominant) |
| 215446_s_at | LOX | lysyl oxidase |
| 216550_x_at | ANKRD12 | ankyrin repeat domain 12 |
| 219729_at | PRRX2 | paired related homeobox 2 |
| 220327_at | VGLL3 | vestigial like 3 (Drosophila) |
| 221916_at | NEFL | neurofilament, light polypeptide 68kDa |
| 222101_s_at | DCHS1 | dachsous 1 (Drosophila) |
| 222572_at | PPM2C | protein phosphatase 2C, magnesium-dependent, catalytic subunit |
| 222662_at | LOC286044 | hypothetical protein LOC286044 |
| 223217_s_at | NFKBIZ | nuclear factor of kappa light polypeptide gene enhancer in B-cells inhibitor, zeta |
| 224567_x_at | MALAT1 | metastasis associated lung adenocarcinoma transcript 1 (non-coding RNA) |
| 225524_at | ANTXR2 | anthrax toxin receptor 2 |
| 225626_at | PAG1 | phosphoprotein associated with glycosphingolipid microdomains 1 |
| 225681_at | CTHRC1 | collagen triple helix repeat containing 1 |
| 226051_at | SELM | selenoprotein M |
| 226810_at | --- | MRNA full length insert cDNA clone EUROIMAGE 1509279 |
| 227484_at | --- | CDNA FLJ41690 fis, clone HCASM2009405 |
| 227974_at | --- | Transcribed locus |
| 228228_at | DACT3 | dapper, antagonist of beta-catenin, homolog 3 (Xenopus laevis) |
| 229218_at | COL1A2 | Collagen, type I, alpha 2 |
| 229222_at | FLJ21963 | FLJ21963 protein |
| 229800_at | DCLK1 | Doublecortin-like kinase 1 |
| 230463_at | --- | CDNA FLJ36891 fis, clone BRACE2000368 |
| 231766_s_at | COL12A1 | collagen, type XII, alpha 1 |
| 235231_at | ZNF789 | zinc finger protein 789 |
| 235248_at | BTBD9 | BTB (POZ) domain containing 9 |
| 239913_at | SLC10A4 | solute carrier family 10 (sodium/bile acid cotransporter family), member 4 |
| 240432_x_at | --- | Transcribed locus |
| 243539_at | KIAA1841 | KIAA1841 |
| 266_s_at | CD24 | CD24 molecule |
