# Supplementary figures and images for: EWS/FLI Mediates Transcriptional Repression via NKX2.2 during Oncogenic Transformation in Ewing's Sarcoma
Source: PLoS One. 2008 Apr 16;3(4):e1965. doi: 10.1371/journal.pone.0001965 (PMC2291578; doi:10.1371/journal.pone.0001965)

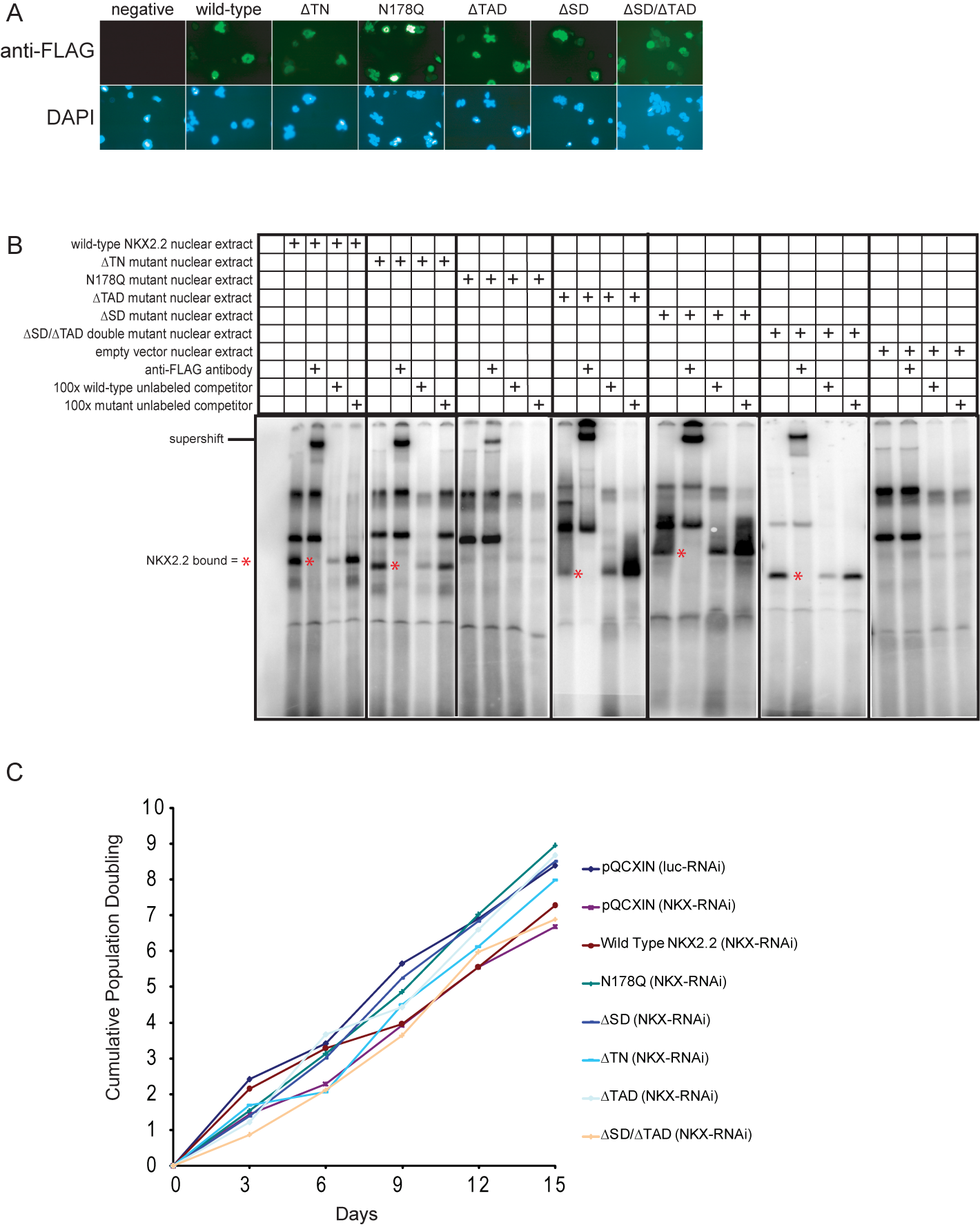

Supplement: Figure S1 — NKX2.2 mutants are localized to the nucleus, bind DNA appropriately, and have growth characteristics similar to control. (A) Immunofluorescence of A673 cells infected with the indicated cDNA constructs with anti-FLAG antibody demonstrate nuclear localization of each of the NKX2.2 mutants. Nuclei are shown by DAPI staining. (B) Electrophoretic mobility shift assays (EMSA) using nuclear extracts from 293EBNA cells transfected with the indicated constructs demonstrate that all of the NKX2.2 constructs, except for the N178Q homeodomain DNA binding mutant, bind a consensus NKX2.2 DNA duplex. Supershifts were performed using anti-FLAG antibody. Specific complexes were competed using wild-type unlabeled competitor, while non-specific complexes were competed using mutant unlabeled competitor. The position of NKX2.2 bound complexes are indicated in the first lane of each group by a red asterisk. (C) Growth analysis using a 3T5 assay demonstrates that A673 cells harboring the NKX2.2 mutant constructs grow similarly to cells expressing the wild type NKX2.2 or empty vector rescue constructs. These data are a representative example from multiple experimental replicates. (7.61 MB TIF) [file pone.0001965.s002.tif]

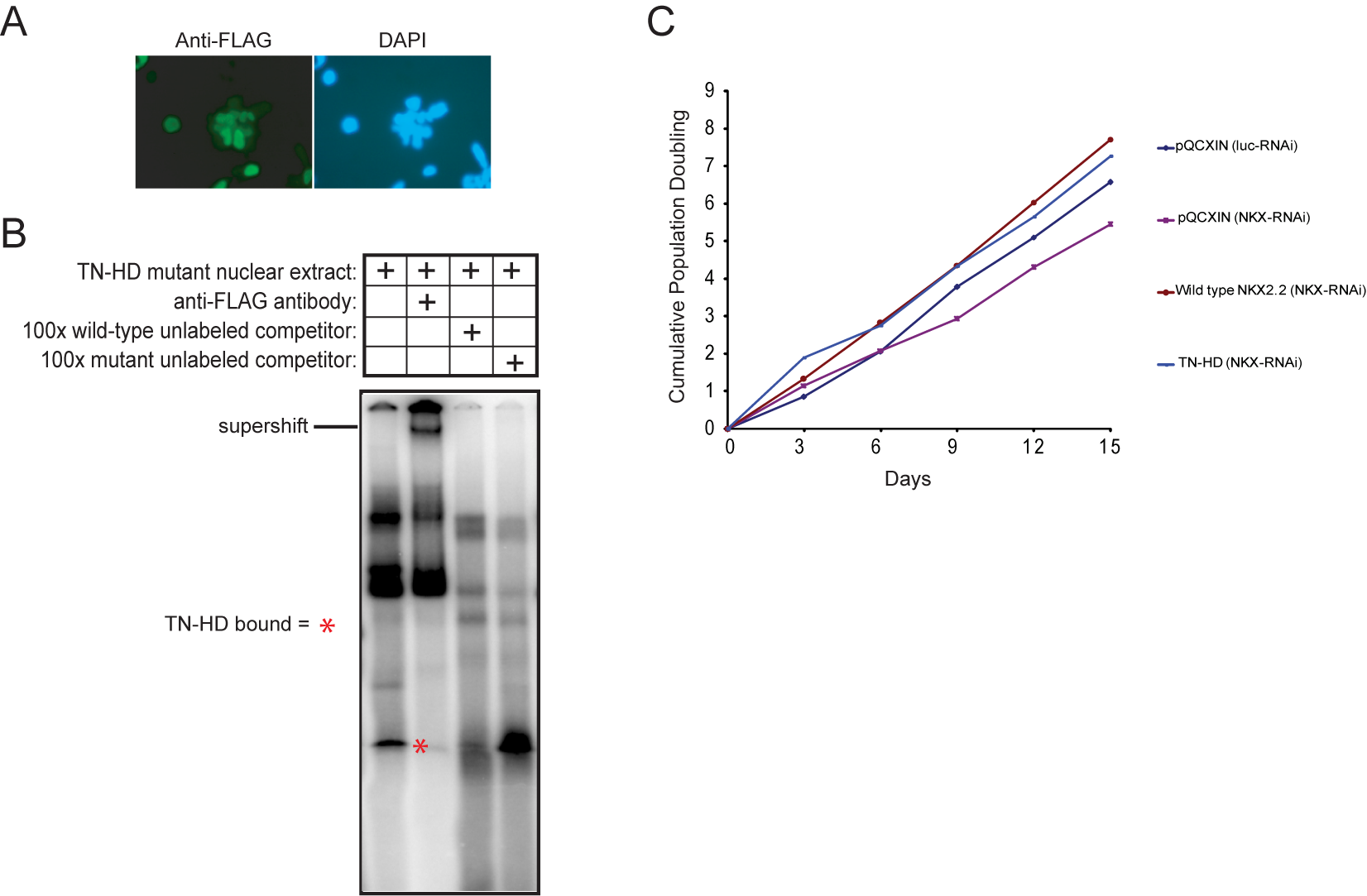

Supplement: Figure S2 — The TN-HD mutant is localized to the nucleus, binds DNA, and has growth characteristics similar to control. (A) Immunofluorescence of A673 cells infected with the indicated cDNA constructs with anti-FLAG antibody demonstrate nuclear localization of the TN-HD NKX2.2 mutants. Nuclei are shown by DAPI staining. (B) Electrophoretic mobility shift assays (EMSA) using nuclear extracts from 293EBNA cells transfected with the TN-HD fusion protein demonstrates that the fusion binds a duplex DNA containing an NKX2.2 consensus binding site. Supershift was performed using anti-FLAG antibody. Specific complexes were competed using wild-type unlabeled competitor, while non-specific complexes were competed using mutant unlabeled competitor. The position of the TN-HD-bound complex is indicated in the first lane by a red asterisk. (C) Growth analysis using a 3T5 assay demonstrates that A673 cells harboring the NKX2.2 mutant TN-HD grows similarly to cells expressing the wild type NKX2.2 or empty vector rescue constructs. These data are a representative example from multiple experimental replicates. (5.39 MB TIF) [file pone.0001965.s003.tif]
